# Crowding analysis for patients with mental disorders during the first pandemic wave of 2019 coronavirus epidemic (CoViD-19) at a lombardy ED

**DOI:** 10.1192/j.eurpsy.2021.973

**Published:** 2021-08-13

**Authors:** G. Savioli, S. Pesenti, I. Ceresa, E. Oddone, M.A. Bressan

**Affiliations:** 1 Emergency Department, IRCCS Policlinico San Matteo, Pavia, Italy; 2 Department Of Public Health, University of Pavia, Pavia, Italy

**Keywords:** metal disorder, COVID-19 pandemic, Emergency department, crowding

## Abstract

**Introduction:**

The 2019 coronavirus epidemic (CoViD-19) in Italy originated in Lombardy, on February 21, 2020. Crowding has been defined as a worldwide problem and causes reduced quality of care. It is due and identified by three orders of factors: those at the access (input); those related to the patient’s process (throughput); and those at the exit from the ED (output).

**Objectives:**

We evaluated all the population who went to ED for mental disorder. Due to the high level of care needed and the simultaneous exposure to risk factors, an excessive duration of ED process can be counterproductive.

**Methods:**

We evaluated all patients accessing our ED for mental disorder from February 22 to May 1, 2020 and during the same period of the previous year.

**Results:**

We enrolled 345 patients. The Crowding input factors are lower in the pandemic period: reduced attenders (142 vs 203) and reduced average waiting times (40 min vs 54 min). The Crowding throughput factors have instead worsened: LOS (length of stay) for both visit rooms (383 vs 271 min) and holding area (1735 min vs 797 min). The Crowding output factors also worsened: the percentage of access block is higher during the pandemic (100% vs 20%). The Total Access Block Time is significantly higher in the CoViD period for both the visit rooms (3.239 vs 649 min) and the holding area (590 vs 185 min).

**Conclusions:**

The pandemic period presented a worsened crowding for these patients due to the Access Block.

